# Spinal Analgesia

**Published:** 1909-07-03

**Authors:** 


### Anæsthetics.

### SPINAL ANALGESIA.

The attitude of the medical profession of this country towards the recently introduced and powerfully advocated methods of obtaining analgesia by the injection of various drugs into the theca spinalis is still one of hesitancy. The leaders of medical thought are still greatly at variance over the advantages and still more over the disadvantages, of spinal injections; and it is not surprising, therefore, that the rank and file should still regard it with a certain degree of suspicion.

Two recent discussion in London on the merits and demerits of the methods used, showed that quite a number of surgeons and anæsthetists, have afforded them a trial. In the experimental stage, which admittedly has not yet been passed, it is of interest to note individual experience and opinion on the various methods of administration, dosage, drug used, posture, and so forth. Mr. Canny Ryall, in opening a discussion on June 4 at the West London Medico-Chirurgical Society, showed at least that possibilities hitherto unthought of are within the range of practical politics, even if it must be added that not all his audience were convinced of the superiority of spinal analgesia over general anæsthesia.

The argument on which his experimental work and its practical applications are based is briefly this:—Difficulties such as faintness, cessation of respiration, and shock, following spinal injection are due to the toxic action of the drug upon the vital centres of the medulla and brain by direct extension in the cerebro-spinal space, not to absorption and subsequent diffusion through the blood stream. This conclusion is based upon experimental work on animals, which has shown that a given dose is more fatal when injected into the spinal theca than when injected into the circulation by venous puncture; it has also been proved that a dose which is fatal when injected into the lumbar spine is not fatal when a ligature has previously been applied round the cord and its membranes at a higher level. Mr. Ryall argues that some means must be found by which the important centres can be protected from this depressing and toxic action. Such a means is afforded, he believes, by the combination of strychnine with the anæsthetic used (novocain in his cases). Now strychnine, when intra-spinally injected, has a very much more powerful effect upon the cerebral nervous system than when given subcutaneously or intravenously, and therefore it becomes important to know precisely what its effects in a given dosage are. It is found that to administer .001 gramme of strychnine into the cervical cerebro-spinal fluid of an adult man or woman has the effect of rendering the patient's nervous system so excitable that a very slight stimulus to any part will throw it into spasm; and half that dose is accordingly considered sufficient to protect the respiratory centre from any danger of depression from the analgesic dose of novocain. When the injection is made in the lumbar or lower dorsal region more strychnine is required, and the full milligramme may then appropriately be introduced.

Protected in this way, he does not hesitate to assert, spinal analgesia may be safely and advantageously used for operations in any region from the crown of the head downwards. For operations on the head, neck, tongue, thyroid, and so forth, the site of puncture is between the axis and atlas vertebræ. Here the cerebro-spinal space is large, and the danger of puncturing the cord is said not to exist provided the cardinal rule is observed of pushing in the needle quite slowly, and of never injecting until a free flow of cerebro-spinal fluid appears through it. The dose recommended for an ordinary adult is .02 to .08 gramme of novocain with .0005 gramme of strychnine in 1 c.c. of water; for children under ten half of these quantities are used. The exact dosage depends on the probable duration of the operation, and on the previous exhibition of scopolamine-morphine; the age of the patient makes but little difference. The patient sits up for two minutes after the injection, and is then put supine, with the head low; there is no objection at all to the Trendelenburg position.

For operations on the arms and thorax, the puncture is made between the fourth and fifth cervical vertebræ, and the patient is allowed to sit up for three minutes instead of two before being placed with the head low. For operations on the liver, stomach, gall-bladder and upper abdomen generally, the puncture is made in the eleventh dorsal interspace, and a somewhat larger dose is given—that is, .05 to .15 gramme of novocain with .001 gramme of strychnine. Here, and in the lower cervical region, the thickness of the layer of fluid between the dura mater and the cord is very much less than it is in the upper cervical vertebræ, and corresponding care to introduce the needle very slowly is essential. For the lower limbs, rectum, pelvis, etc., the dose of novocain is from .06 to 0.2 gramme with 1 milligramme of strychnine. This is introduced in the first lumbar space, or lower. It is very important to keep most accurately in the middle line, and to inject into the median space, some 2 to 6 millimetres in width, which separates the bundle of the cauda equina of one side from that of the other; otherwise the injected fluid may become enmeshed in one or other bundle, and a unilateral or unsatisfactory anæsthesia may result.

Mr. Ryall feels very strongly that by this combination of strychnine with novocain a definite gain is obtained, and a very grave objection to spinal analgesia avoided. In the discussion which followed it was evident that those who have had experience of spinal analgesia are unfamiliar with the strychnine combination, and are therefore not in a position either to criticise or to belaud it. Broadly speaking, the surgeons are much more enthusiastic in their advocacy of spinal methods than are the anæsthetists. It is especially notable what a large proportion of the former expressed the opinion that the dangers of general anæsthesia are very gravely underestimated. It is, perhaps, only natural that surgeons, taken as a whole, should be impressed more than are anæsthetists by the frequency of post anæsthetic trouble, such as headache and vomiting, because to them falls very often the treatment of such symptoms. Indeed, the anæsthetist may never hear of their occurrence. There is an undoubted tendency to speak as if these troubles are the rule instead of the exception, which is certainly the case after the administration of a general anæsthetic by a reasonably practised administrator.

At the present time and in the present state of knowledge it is not unfair to sum up the situation in regard to spinal analgesia thus. The technique requires considerable practice and the most perfect comprehension of asepsis. In practised hands it is nearly always successful in producing complete analgesia and a degree of relaxation which is eminently satisfactory to the operator. It is especially indicated for prostatectomy, an operation demanding relaxation which it is both difficult and dangerous to get by inhalation methods, and one often performed on those for whom on every ground a general anæsthetic is highly inadvisable. For those with serious disease of the lungs or kidneys, also, it is strongly indicated; but it is as unquestionably contra-indicated for those who are the subject of any general infection, as suppurative meningitis not uncommonly then follows. Its dangers, immediate and remote, are not yet fully ascertained; but they are distinctly not a negligible quantity. Several deaths have occurred, and other sequelæ, less serious but decidedly unpleasant, are not rare. Thus a considerable proportion of patients suffer from headache, which may last several days, and ocular paralyses are among the most recently reported mischances.* On the whole the method cannot as yet be recommended to general practitioners, for many of those who have tried it are still unconvinced of its superiority to ether and chloroform; but it seems possible that improvements in administration may widen the scope of its usefulness, and that it may still prove a formidable rival to those drugs. As far as matters have gone at present this stage has not yet been reached.

*See report of 12 such cases in the *British Medical Journal*, June 5, 1909.

